# How to evaluate first aid skills after training: a systematic review

**DOI:** 10.1186/s13049-022-01043-z

**Published:** 2022-11-08

**Authors:** Sihvo Minna, Hiltunen Leena, Kärkkäinen Tommi

**Affiliations:** 1 Faculty of Information Technology, University of Jyväskylä, Jyväskylä, Finland; 2 Punainen Risti Ensiapu (Red Cross First Aid), Helsinki, Finland

**Keywords:** First aid training, Evaluation method, Systematic literature review

## Abstract

**Background:**

To be able to help and save lives, laypersons are recommended to undergo first aid trainings. The aim of this review was to explore the variety of the elements of the measuring systems to assess the effects of first aid trainings on different aspects of first aid skills including practical skills, knowledge, and emotional perspectives.

**Methods:**

This systematic literature review used Scopus and PubMed databases and searched for studies published between January, 2000, and December, 2020. Out of 2,162 studies meeting the search criteria, 15 studies with quantitative and repeatable evaluation methods to assess first aid skills after first aid training for adults were included in the final analysis.

**Results:**

Practical skills, especially on the ability to perform cardiopulmonary resuscitation (CPR) and to use an automated external defibrillator, were the most studied first aid skills after first aid training. This evaluation was based on several standardized measurements and assessed often with the help of a combination of resuscitation manikin and observer. Evaluation methods of performance in other emergency situations are not well standardized. Questionnaires used to assess knowledge of first aid, though seemingly based on guidelines, were also not standardized, either. Emotional aspects of first aid (willingness or self-confidence) were evaluated by highly simplified questionnaires, and answers were graded by five-point Likert scale.

**Conclusion:**

According to our review, the focus of evaluation methods after first aid training has been on practical skills and especially on CPR. Though the evaluation of first-aid knowledge seems to be straightforward, it is not performed systematically. Evaluation methods for emotional aspects are highly simplified. Overall, standardized measurements and evaluation methods to assess all aspects of first aid skills are needed.

**Supplementary Information:**

The online version contains supplementary material available at 10.1186/s13049-022-01043-z.

## Introduction

Out-of-hospital cardiac arrest (OHCA) cause 350,000 deaths each year in Europe [1]. In Finland, the annual incidence of OHCA is typically 46–80/100,000 inhabitants [2]. In most circumstances, survival rates are low being just above 10% [2]. With the help of a layperson, these survival rates could, however, be doubled [3]. Indeed, the cardiopulmonary resuscitation (CPR) given by laypersons has likely increased the survival rate of patients who underwent OHCAs during the past decades [4]. The first person to witness an OHCA, therefore, plays a critical role in the survival of a person having a cardiac arrest [5, 6]. Stroke, with an annual incidence the annual number of approximately 14,000 in Finland, is one of the leading causes of death and disability [7]. The early detection of stroke in the prehospital setting has the potential to decrease delays in treatment and, hence, improve stroke outcomes [8, 9]. Therefore, awareness of stroke warning signs by a layperson is important.

In Europe (EU-27), 146,000 people died in over 4.5 million accidents (mortality rate, 3.2%) in 2016 [10]. The number of accidents in Finland with 5.5 million inhabitants caused by a physical injury at home or leisure time is more than a million [11]. The mortality of these accidents is higher in Finland (4.7%) than in other Nordic countries and this figure is approximately 1.5 times the EU average [10]. Overall, first aid skills enabling one to cope with all common emergency situations, in addition to OHCA, are needed [7, 11].

According to European guidelines [12], the goals of first aid include preserving life, alleviating suffering, preventing further illness or injury, and promoting recovery. First aid, defined as the initial care provided by a layperson for an acute illness or injury, has been shown to save lives and to reduce morbidity [3, 13, 14]. To be able to act in any emergency situations, laypersons have undergone various kinds of first aid trainings [15–18]. A typical setting for a training course is a traditional on-site class-room training, an on-line course, or a combination of these two (blended learning). Modern technologies, such as virtual techniques, smartwatches, and mobile devices, can be used to enhance any of these training sessions [19–25]. Although, millions of people have been trained each year with some evidence that these people have a greater tendency to perform CPR in a real-world situation [6, 26], the overall efficacy of first aid training is not clear [27, 28].

In first-aid trainings, knowledge and practical skills are easy to teach. In a sudden life-threatening situation, human factors as self-efficacy, willingness to help, and courage, are also meaningful [29, 30]. Despite undergoing first aid training, people often have low confidence to help before professional assistance arrives [29, 31, 32]. The effect of first aid training on theses human factors is poorly studied [16, 29]. Studies have concentrated especially on the practical skills of performing CPR and the use of an automated external defibrillator (AED) [3, 13, 16, 18]. Other practical first aid skills, such as required in choking, hemorrhage, stroke, and trauma have been less focused on [33].

Measuring the knowledge and behavior of a helper for all aspects of first aid skills is challenging. Whether an optimal quality of CPR can be defined has even been questioned [3]. Despite the numerous studies of first aid trainings, the lack of standardized measurements to evaluate all aspects of first aid skills is evident. The aim of this review was to explore the variety of the elements of the measuring systems to evaluate different aspects of first aid skills including practical skills, knowledge, and emotional perspectives.

## Materials and methods

This systematic review was following Preferred Reporting Items for Systematic Reviews and Meta Analyses (PRISMA) 2009 guidelines [34]. Because the aim was to explore the variety of methods used in the evaluation of first aid training effect and not to analyze the study outcomes, some parts of the PRISMA 2009 checklist were not applicable (see Additional file 1). Studies with adult participants in the first aid training were included, which composed the population (P) in the Population, Intervention, Comparator, Outcome (PICO) question.

### Eligibility criteria

To inspect the elements used to assess first aid training effect, we used the following inclusion criteria:

1. Participants were 18 years or older without health education background.
2. Studies were written in English and published between 2000 and 2020.
3. Studies followed the guidelines published either by the American Heart Association (AHA) [35] or European Resuscitation Council (ERC) [12].
4. The training effect on practical skills, knowledge and/or emotional aspects were evaluated with an explicitly specified evaluation method that is repeatable and provides a quantitative outcome.

We concentrated on the studies of first aid for acute illnesses and traumas. Therefore, studies of mental health first aid were excluded. Due the special feature of neonatal or infant first aid, these studies were also excluded. All studies from countries with limited access to quality health care and education were also excluded because first aid training system and the required skills can differ between these and those countries included.

### Information sources and search strategy

All possible studies were retrieved either from Scopus or Pubmed. Database search started on September 2020 and ended on December 2020 and included studies published between January 1, 2000, and December 31, 2020. Used keywords first in Scopus and then in Pubmed were as follows: first aid AND layperson(s), first aid AND bystander(s), first aid AND helper(s), first aid AND learning, first aid AND teaching, first aid AND education, first aid skill(s). During the Pubmed search, duplicates were excluded. The number of studies, meeting the search criteria in this first step of the selection process was 2,163.

### Study selection process

**Fig. 1:**
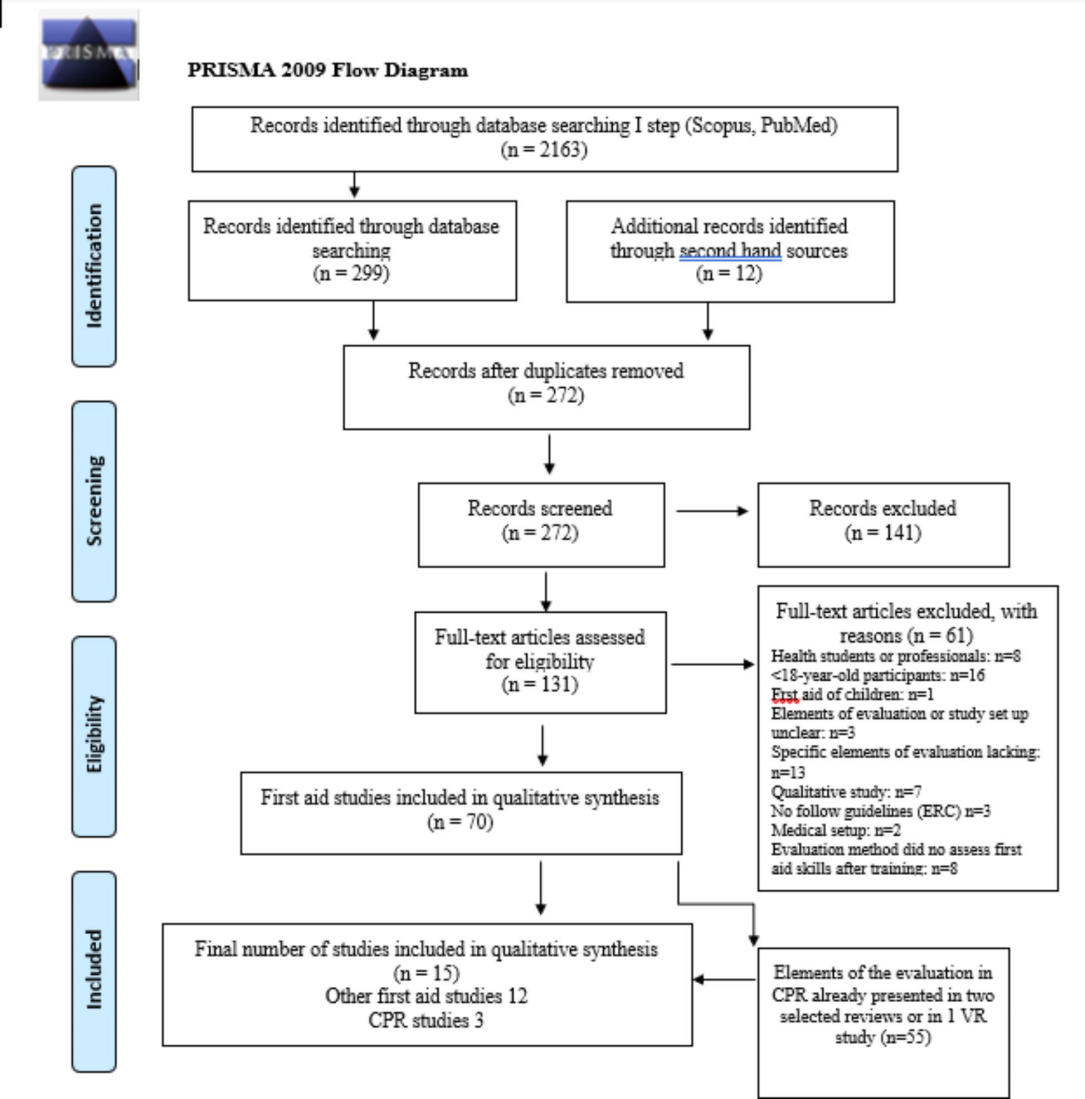
**Flow diagram of included studies. ** Adapted from: Moher D, Liberati A, Tetzlaff J, Altman DG, The PRISMA Group (2009). Preferred Reporting Items for Systematic Reviews and Meta-Analyses: The PRISMA Statement. PLoS Med 6(7): e1000097. doi:10.1371/journal.pmed1000097

Those 2,163 studies meeting the search criteria were further screened by the first author. Because this review was not an outcome analysis, excluding studies due to quality issues was unnecessary. It was more important to able to identify all possible sources of the evaluation methods under study. The whole process is shown in the flow diagram in Fig. 1. Based on the abstracts and titles of the studies, 299 studies were included in further evaluation. The references of these 299 studies were also checked, yielding 12 additional studies. After duplicates were removed, the number of studies was 272. After the evaluation of full-text articles and exclusion of those 141 studies not meeting the earlier described inclusion criteria, the number of included studies was 131.

In the detailed evaluation, 61 additional studies were excluded based on the eligibility criteria (Fig. 1): (1) Participants in the study were health care students or professionals (n = 8) or < 18-year-old (n = 16), (2) study concentrated the first aid of children (n = 1), (3) the study setup or the elements to measure training outcomes were unclear (n = 3), lacking (n = 13), qualitative (n = 7) or did not follow guidelines (ERC or AHA) (n = 3), (4) the study was a part of a medical study (n = 2) and (5) evaluation method did not assess first aid skills after training (n = 8). All excluded studies are listed in Additional file 2.

Of the 70 studies that were found eligible, 58 studies on CPR and AED and 12 studies on other aspects of first aid remained to be evaluated in the qualitative synthesis. It became, however, soon evident that those 58 included CPR studies repeatedly applied the same evaluation methods to assess the training effects. Because the interest of the study was in the variety of elements to measure the first aid training effect, we started an additional evaluating process about CPR studies to simplify the description of the results in this synthesis. In this process, we compared the evaluation methods used in a single CPR study to the evaluation methods described in two very recent CPR reviews [13, 16]. If these reviews already included the used elements of the evaluation systems, a study was excluded. All excluded CPR studies according to this additional evaluation are listed in Additional file 3. The total number of studies whose evaluation methods were included in the reviews, and therefore excluded, was 55. Therefore, of the 58 CPR studies, only these two reviews and a single study using virtual reality (VR) technology [19] were included in the final analysis. Overall, the number of studies that underwent final detailed evaluation and qualitative synthesis was 15. These studies are depicted in Table 1.

**Table 1:** Characteristics of selected studies

Authors	Article	Pubiliction	Year	Scope
Baruch (Israel)	Does practice make perfect? Prospectively comparing effects of 2 amounts of practice on tourniquet use performance	American Journal of Emergency Medicine	2016	Bleeding
Goolsby et al. (USA)	Brief, Web-Based Education Improves Lay Rescuer Application of a Tourniquet to Control Life-Threatening Bleeding	AEM Education and Training	2018	Bleeding
Goralnick et al. (USA)	Effectiveness of Instructrional Interventions for Hemorrhage Control Readiness for laypersons in the Public Access and Tourniquet training Study (PATTS). A Ranadomized Clinical Trial	Jama Surgery	2018	Bleeding
Goolsby et al. (USA)	Layperson Ability and Willingness to Use Hemostatic Dressings: A Randomized, Controlled Trial	Prehospital Emergency Care	2019	Bleeding
McCarty et al. (USA)	Can they stop the bleed? Evaluation of tourniquet application by individuals with varying levels of prior self-reported training	Injury	2019	Bleeding
Scott et al. (USA)	Ability of Layperson Callers to Apply a Tourniquet Following Protocol-Based Instructions From an Emergency Medical Dispatcher.	Prehospital Emergency Care	2020	Bleeding
Watson & Zhou (USA)	BreathEZ: Using smartwatches to improve choking first aid	Smart Health	2018	Choking
Watson & Zhou (USA)	BBaid: Using smartwatches to improve back blows	Smart Health	2019	Choking
Handschu et al. (Germany)	The Challenge of community-Based Research. The Beauty Shop Stroke Education Project	J Neurol	2006	Stroke
Kleindorfer et al. (USA)	First Aid in acute stroke. Introducing a concept of first action to laypersons	Stroke	2008	Stroke
Ertl & Christ (Germany)	Significant improvement of the quality of bystander first aid using an expert system with a mobile multimedia device	Resuscitation	2007	Multiple scenarios
Anderson et al. (Canada)	First aid skill retention of first responders within the workplace	Scand J Trauma Resusc Emerg Med	2011	Multiple scenarios
Chen et al. (Taiwan)	Interventions to improve the quality of bystander cardiopulmonary resuscitation: A systematic review	PLOS One	2019	CPR
Riggs et al. (Australia)	Associations between cardiopulmonary resuscitation (CPR) knowledge, self-efficacy, training history and willingness to perform CPR and CPR psychomotor skills: a systematic review	Resuscitation	2019	CPR
Buttusi et al. (Italy)	A virtual reality methodology for cardiopulmonary resuscitation training with and without a physical mannequin	Journal of Biomedical Informatics	2020	CPR

## Results

Of the 15 analyzed studies, two reviews [13, 16], and one randomized trial [19] provided the evaluation methods to assess resuscitation skills (Table 1). The randomized study not included in these two reviews used modern virtual technology both in training and in the evaluation process and was, therefore, relevant. Twelve studies provided those evaluation methods to assess first aid skills other than CPR [15, 25, 27, 36–44].

Those included studies were classified into three separate groups based on which aspect of first aid skills they deal with: knowledge, practical skills, or emotional aspects (self-confidence, courage, willingness). Studies measuring more than one aspect of first aid skills were included in all relevant categories (Table 2). Studies included in those two CPR reviews and the only separate randomized CPR study were classified accordingly but discussed in a separate section.

**Table 2:** Overview of the studies and the evaluation methods used to assess aspects of first aid

	Baruch et al. (2016)	Goolsby et al. (2018)	Goralnick et al. (2018)	Goolsby et al. (2019)	McCarty et al. (2019)	Scott et al. (2020)	Watson & Zhou (2018)	Watson & Zhou (2019)	Handschu et al. (2006)	Kleindorfer et al. (2008)	Ertl & Christ (2007)	Anderson et al. (2011)
**Study design**												
RCT	RCT	RCT	RCT	RCT	RCT	RCT		RCT			RCT	RCT
Cohort							Cohort		Cohort	Cohort		
Total number of participant	156	269	465	360	317	246	10	16	532	383	101	257
Scenario or subject of first aid	Bleeding	Bleeding	Bleeding	Bleeding	Bleeding	Bleeding	Choking	Choking	Stroke	Stroke	Multiple scenarios	Multiple scenarios
**Timing of the testing**												
Before intervention	X						X	X	X	X		
< 1 month after intervention			X	X		X	X	X	X		X	X
1–3 months after interventions		X								X		X
> 3 months after interventions	X		X		X					X		X
**Practical skills of first aid**												
Recorded by observer		X	X	X	X	X		X			X	X
Recorded by manikin or digital device	X			X		X	X	X				X
**Knowledge on first aid**												
Scenario based/open ended questions		X							X	X		
Multiple choice questions										X		X
**Emotional aspects of first aid**												
Scale 1–5 Likert		X		X	X		X	X				
Interview											X	

### Practical skills of first aid

Of 12 non-CPR studies, 10 uncovered practical skills (Table 2). Practical skills in all these studies were evaluated in different experimental first-aid situations (Table 3).

**Table 3:** Overview of the elements used to assess practical first aid skills in various scenarios

Practical skills of first aid			
**Scenario**	**Study**	**Recorded by the observer**	**Recorded by the manikin or by digital device**
**CHOKING**	Watson et al. (2018)	Calling 911, position of the manikin	Smartwatch: Maximum acceleration of back blow
	Watson et al. (2019)		Smartwatch: Acceleration and number of back blows
**TOURNIQUET USE**	Scott et al. (2020)	Recorded manually digital data for backup	HapMed leg Tourniquet Trainer: the total time needed to stop bleeding (< 4 min), tourniquet placement (2–3 in.), tourniquet pressure (> 200 mmHg), estimated blood loss (< 2500 mL), pressure status (good, loose or tight)
	Goolsby et al. (2018)	Correct tourniquet application: position, pressure (adequate if observer unable to slide finger under the band), time (< 7 min), reasons of failure.	
	Baruch et al. (2016)		HapMed leg Tourniquet Trainer: application time in seconds (start/done-point) and tourniquet pressure (> 200 mmHg)
	McCarty et al. (2019)	Correct tourniquet application: distance above injury (2 inc), adequate tightness (observer unable to slide thin instrument between tourniquet and mannequin), time (< 7 min).	
	Goralnick et al. (2018)	Correct tourniquet application: distance above injury (2 inch), adequate tightness (observer unable to slide thin instrument between tourniquet and mannequin), time (< 7 min).	
**HAEMOSTATIC DRESSING**	Goolsby et al. (2019)	Correct hemostatic dressing location Reasons for failure	Z-medica simulator: sufficient pressure (250 mmHg)
**MULTIPLE SCENARIOS**	Anderson et al. (2011)	Checklists: scene safety, airway, airway clearance (abdominal thrust), emergency call activation, airway opening, ventilation trying	Recording manikin: rate, depth, frequency of ventilation and rate, depth and location of chest compressions
	Ertl & Christ (2007)	OSCE (Objective Structured Clinical Examination) two scenarios (CPR and severe bleeding)	

### Choking

Two studies [25, 41] assessed first aid skills in an experimental choking situation. The data for the assessment were provided either by an application and an observer or only by a smart watch application. A smartwatch was worn by a participant while performing first aid for choking on a CPR manikin. The quality of back blow or abdominal thrust was evaluated by a quantitative accelerometer and gyroscope data provided by the smartwatch. According to these data, the BreathEZ application, in both studies, classified the quality of back blow or abdominal thrust as too soft, correct, or too hard. In back blows, observer also marked whether simulated 911 calling and the correct manikin position were done properly.

### Tourniquet use

Of five bleeding control studies, the observer inspected the application of a tourniquet in four [36, 37, 39, 40]. In two HapMed leg Tourniquet Trainers provided additional digital data [15, 40]. In one study [15], the outcome was based only on the data provided by the recording manikin. The roles of the observer in these studies were (1) to confirm all successfully completed digital recordings, (2) to verify correct tourniquet application, and (3) to assess the participant`s performance during the application.

The assessment of tourniquet application included the evaluation of (1) positioning, (2) tightness using a thin instrument or a finger between the tourniquet and the mannequin, (3) the steps required to complete the application, and (4) the time of application with the aim being less than 7 min (Table 3). The recording manikin and HapMed leg Tourniquet Trainer also provided data on time scale and pressure status.

### Hemostatic dressing

The only study of the use of hemostatic dressings in first aid relied on the subjective data provided by the observer and the digital data provided by a Z-medica simulator [38]. This simulator provided exact feedback only of the amount of pressure applied. The defined pressure limit for the correct application of the hemostatic dressing was 5 pounds per square inch, approximately 250 mmHg. The required time to apply the dressing was a maximum of 3 min. The technical performance was graded by the observer either success or failure based on the checklist modified from the existing AHA´s first aid/CPR/AED recommendations [38]. If the participant made a mistake in any step of the application, it was scored as a failure.

### Multiple scenarios

In a choking scenario causing a cardiac arrest, both the first aid of choking and resuscitation were evaluated [27]. The evaluation process relied on the data provided by the observer and recording manikin. The observer assessed the correct order and proper execution of each step (scene safety, unconsciousness, clearing of airways with abdominal thrusts, calling emergency medical services, opening of airway, and taking care of breathing). The recording manikin measured the same parameters as in resuscitation studies: the rate, depth, and frequency of breathing and the rate, depth, and the location of chest compressions.

The other study evaluated practical first aid skills to control severe bleeding and to apply CPR [44]. The evaluation of the performance was based on a scoring tool—the Objective Structured Clinical Examination (OSCE). In OSCE, actors play planned scenarios. Specially trained examiners assessed the performance according to the pre-defined evaluation criteria. A maximum of 24 points could be achieved. In the first station, 13 different actions to control severe bleeding were scored; in the second, 11 required actions in the resuscitation were scored.

### Knowledge on first aid

First aid knowledge in acute stroke has been evaluated in two studies [42, 43]. A study by Handschu et al. explored whether a short 15 to 20 min session will increase the general knowledge of stroke and the recognition of stroke symptoms [42]. The other study [43] assessed how well warning signs and risk factors of stroke are identified 6 weeks and 5 months after intervention. In a both studies, data were collected by open-ended questions. Multiple-choice questions were used only in the study by Kleindorf et al. [43].

Scenario-based questions have been used to assess the level of first aid knowledge in case of massive bleeding [36]. Scenarios were based on pictures of extremity wounds to decide of the need of a tourniquet. One study tested first aid knowledge by a multiple choice first aid exam using questions from the Worker’s Compensation Board of British Columbia´s first aid exam [27].

### Attitudes and emotional aspects of first aid

Emotional aspects have been included in studies of choking, bleeding control, and multiple first aid scenarios. These studies scaled the comfortableness and the willingness to help in given scenarios from 1 to 5. In a study by Ertl and Christ [44], attendants were interviewed to evaluate the most important causes of stress in an emergency situation and subjective requirements to eliminate this kind of stress factor. The answers were categorized as follows: no stressor, lack of skill, fear of being alone, fear of doing harm, strain after having help, revulsion, findings on patient´s status, being self-responsible, and pressed for time.

### Measuring practical skills, knowledge, and emotional aspects of resuscitation

Resuscitation or basic life support-skill (CPR/AED) is the most studied area of the field first aid skills. In many of these trials, the setting is a randomized control study [13, 16]. These studies have been conducted without intervention, before and after intervention, within a month after intervention, within 1–3 months after intervention, and more than 3 months after intervention.

### Practical skills

The quality of BLS-skills (CPR/AED) have been measured by a recording manikin, a recording simulator manikin, a manikin with special software, and a recording defibrillator [13, 16]. A combination of observer’s subjective evaluation, a recording video, and a recording manikin has also been used. Testing after new VR technology-based resuscitation training has relied on the data provided by the sensors inside the mannequin or on the wrists [19]. With the webcam (focused on the mannequin), it was possible to confirm the quality of CPR.

Despite of evaluation method, the quality of CPR was based on the compression rate (100–120/min), compression depth (50–60 mm), compression-ventilation ratio, duration of interruptions between chest compressions, full chest recoil, correct hand placement, the amount of correct compression, ventilation volume (500 ml), ventilation time (s), and overall performance. Overall performance was estimated by using a modified checklist filled by the observer. The checklist was adopted either from the AHA`s guidelines or from the ERC guidelines [13, 16]. They include the following parameters: safety check, shoulder shaking, calling for help, head rotation, airway control, breath control, and using AED. AED performance were measured as follows: switching on the AED, removing clothing, time from the start of assessment to switching on the AED, electrodes attached correctly, location of wrongly placed electrodes, time from starting assessment to attaching electrodes, rescuer`s position during analysis, pushing the shock button as directed, shock safety, time from starting assessment to the first shock, and restarting CPR.

### Knowledge, attitudes, and emotional aspects of resuscitation

One of the systematic reviews contained depiction on how the knowledge of resuscitation was measured [16]. Knowledge was measured by open-ended questions and multiple-choice question of clinical scenarios. Willingness, self-confidence, and changes in self-efficacy were measured using a five-point Likert-type scale.

## Discussion

According to our review, most studies assessing first aid skills after the corresponding training have focused on evaluating either practical skills or the knowledge of first aid or both (Table 2). In these studies, CPR is overrepresented. In recent years, emotional aspects, such as willingness to help and self-confidence, have gained interest as well [29]. Despite the large number of studies, the measurement systems used to evaluate first aid skills are not consistent. Only with congruent evaluation methods in all aspects of the first aid skills, we are able compare the outcomes of various first aid trainings held by different organizations including private, non-governmental or governmental organizations.

### Measuring practical skills of first aid

First aid is often seen as a practical skill that can only be learned through practical exercises in created real-life scenarios. Emergency situations played by actors and based on OSCE are the most complex and the closest possible real-scenario where the first-aid skills can be evaluated. This kind of set-up is suitable for research purposes, but its’ role in everyday life after any kind of first-aid training is not a reality. Because the real-life performance in an emergency situation is difficult to measure, the effects of first aid training, especially CPR, are widely evaluated by the resuscitation training manikins. Other devices have also been used in first aid studies to provide real-time performance feedback. HapMed leg Tourniquet Trainer or Z-medica simulator provides measurable digital data of hemostatic effect of a tourniquet or hemostatic dressing [15, 40]. A smartwatch application has been developed to evaluate the effectiveness of a back blow in a choking situation. [25]. Standard manikins have been developed to teach and to measure CPR skills. In some studies, these same manikins are utilized to enhance teaching and the evaluation process in other emergency situations, such as choking or major bleeding [15, 25].

Digital devices give exact digital feedback to analyze the outcomes of learning or teaching [13, 16, 18, 45]. Especially in the evaluation of CPR, a device provides highly specific and detailed and repeatable digital data on parameters, such as compression rate, hand placement, and compression depth [13, 16]. For this evaluation process, such new and more expensive technology as VR have been used, as well [22, 45]. In the implementation of any new and especially costly technology or devices, we have to keep the focus on the aim of first aid teaching. At this point, modern technologies, such as a CPR manikin with digital feedback, provide mostly just more exact data during the first aid training and in the evaluation of training effect. In the near future, modern technology can most likely be utilized widely in both teaching and evaluation of first aid.

Although digital devices provide measurable, repeatable, and comparable parameters of a specific practical skill, the importance in teaching and evaluation of the emergency situation as a whole should be pointed out. Therefore, in many studies included in this review, the evaluations were performed by both an observer and a device and more rarely only by any device (Table 3). For this kind of evaluation, especially for CPR and using AED, the international guidelines provide recommendations and standardized checklists [46, 47]. Checklists have, however, been modified in many CPR studies from these standardized checklists [16, 38]. Although the evidence of using a checklist during the evaluation is inadequate [48, 49], the wide-scale use of a congruent checklist would provide researchers and first-aid instructors data to compare different types of interventions. These recommendations and checklists concentrate on CPR, and the need for those in other emergency situations is evident.

### Measuring knowledge on first aid theory

After first aid training, questionnaires to evaluate knowledge have been used as the sole evaluation method to measure the level of first aid skills. The level of knowledge was evaluated by either multiple-choice, open-ended, or scenario-based questions. This kind of assessment is, however, most often combined to the evaluation of practical skills [13, 16, 36]. In this review, only two first aid training studies to enhance stroke awareness used solely either open-ended questions or multiple-choice questions to assess the improvement in the ability to recognize risk factors, warning signs, and symptoms of stroke [42, 43]. The challenge is that the level of knowledge assessed by these questionnaires does not seem to indicate how well the layperson can perform first aid even in a simulated environment [50]. At the community, the theoretical knowledge on the basics in CPR is poor [51]. Overall, a certain level of first aid knowledge is a requirement to enable to provide optimal help in any emergency situation and to evaluate the level of knowledge after training we need these tests.

The assessment of knowledge between studies has not been congruent. In many studies, researchers have created their own questionnaires [6, 50, 52]. It would seem to be useful to create congruent ways to test theoretical knowledge in different aspects of first aid. With congruent testing, the outcome evaluation of various types of interventions would be comparable and would most likely provide more accurate information. This is especially important during the last years when online learning has increased rapidly.

### Measuring the emotional aspects of first aid

The vital role of first aid is effective only if the layperson, in addition to practical skills and knowledge, has the willingness and confidence to help. Many barriers exist to prevent laypersons to start CPR [53–55] and, therefore, regardless of first-aid interventions the rate of performed CPR if needed in real-life situation remains too low [31, 32]. Out-of-hospital cardiac arrest survival is influenced by human factors [56]. The number of studies evaluating the emotional aspects of first aid is, however, limited. In several studies of this review, willingness or self-confidence were evaluated by a simple questionnaire and answers graded by a five-point Likert scale before and after training [25, 36, 38, 39, 41, 44]. The question is whether this kind of simple assessment is enough to scale the motivation, willingness, and self-confidence after training so that it correlates with the ability to act in a real-life situation.

The ERC [57] has emphasized the importance of emotional and behavioral aspect in teaching first aid. The evaluation of emotional changes after training should also be optimized to enhance this. The ultimate aim would be to improve laypersons’ response rate in any emergency situation.

### Limitations

In this study, some limitations must be considered. First, this systematic review has followed Preferred Reporting Items for Systematic Reviews and Meta Analyses (PRISMA) 2009 guidelines [34]. During the process of this study in 2021, an updated version of PRISMA guidelines were published [58]. The search process of this review had already been done before the updated PRISMA version, and, therefore, it was natural to continue according to the previous version. Second, only two databases were used, and only one person performed the database searches. This does not, however, limit the number of studies included and, therefore, to cause selection bias. Also, two other authors were involved in the study selection throughout the process checking and validating all potential articles. Third, in this review, we did not perform any qualitative analysis. It was, however, our aim to explore the variety of the evaluation methods used in the assessment of first aid skills after first aid interventions.

## Conclusion

According to our review, the focus of first aid interventions and, especially, evaluation methods, has been on CPR. For CPR, the international guidelines provide clear recommendations and standardized checklists to unify the training and the outcome evaluation. The evaluation methods in practical skills other than CPR are not standardized and less used. Repeatable questionnaires are typically utilized to evaluate first aid knowledge. These questionnaires are, however, not congruent, either. Though first aid skills are composed of theoretical knowledge, practical skills, and a courage to help, emotional aspects have gained the least attention in the training and evaluation. We need more information of these emotional factors preventing laypersons to help in a life-threatening situation, and on how trainings can be improved so that a helper is able to overcome fear and anxiety.

A topical question is how the development of online learning and use of modern technology could improve the first-aid trainings [59, 60]. Results from [61] showed that an online course before CPR did improve theoretical knowledge but did not affect practical skills or willingness to help, which was even not influenced by existing emergency experience [62]. However, in order to analyze and compare different modes of training, we need congruent, consistent and transparent methods to evaluate the learning outcomes. Analysis and empirical evaluation of the ability to act in real emergencies is challenging. Only through evidence of the changes in learner´s first aid knowledge, practical skills and self-confidence can we improve the effectiveness of first aid education keeping the ultimate aim in mind – to save more lives.

## Electronic supplementary material

Below is the link to the electronic supplementary material.

Supplementary Material 1

Supplementary Material 2

Supplementary Material 3

## Data Availability

Data in systematic reviews consists of original articles with copyright. Full articles that where analyzed during the systematic review process and are not open access can be requested from the co-author (MS).

## List of abbreviations

CPR: Cardiopulmonary resuscitation
OHCA: Out-of-hospital cardiac arrest
AED: Automated external defibrillator
PICO: Population, Intervention, Comparator, Outcome
AHA: American Heart Association
ERC: European Resuscitation Council
VR: Virtual reality
RCT: Randomized control trial
OSCE: Objective Structured Clinical Examination
