# Supplementary material for: How to evaluate first aid skills after training: a systematic review
Source: Scand J Trauma Resusc Emerg Med. 2022 Nov 8;30:56. doi: 10.1186/s13049-022-01043-z (PMC9641962; doi:10.1186/s13049-022-01043-z)
Supplement: Supplementary file 2 — Supplementary Material 2 [file 13049_2022_1043_MOESM2_ESM.docx]

# Additional file 2. Reasons for excluding full-text articles.

## Health students or professionals

1. Aksoy E. Comparing the Effects on Learning Outcomes of Tablet-Based and Virtual Reality-Based Serious Gaming Modules for Basic Life Support Training: Randomized Trial. JMIR Serious Games. 2019;7:e13442.
2. AlSabah S, Al Haddad E, AlSaleh F. Stop the bleed campaign: A qualitative study from our experience from the middle east. Ann Med Surg (Lond). 2018;24:67-70.
3. Chien C, Weng M, Hsu S, Kuo C-W, Chaou C-H. Effect of population-based training programs on bystander willingness to perform cardiopulmonary resuscitation. Signa vitae. 2016;12:63-69.
4. González-Salvado V, Abelairas-Gómez C, Peña-Gil C, Neiro-Rey C, Barcala-Furelos R, José Ramón González-Juanatey JR, et al. Basic life support training into cardiac rehabilitation programs: A chance to give back. A community intervention controlled manikin study. Resuscitation. 2018; 127: 14-20.
5. Pelinka LE, Thierbach AR, Reuter S, Mauritz W. Bystander trauma care—effect of the level of training. Resuscitation. 2004; 61: 289-296.
6. Semeraro F, Taggi F, Tammaro G, Imbriaco G, Marchetti L, Cerchiari EL. iCPR: a new application of high-quality cardiopulmonary resuscitation training. Resuscitation. 2011;82:436-41.
7. van Dawen J, Vogt L, Schröder H, Rossaint R, Henze L, Beckers SK, et al. The role of a checklist for assessing the quality of basic life support performance: an observational cohort study. Scand J Trauma Resusc Emerg Med. 2018; 26: 96.
8. González-Salvado V, Fernández-Méndez F, Barcala-Furelos R, Peña-Gil C, González-Juanatey JR, Rodríguez-Núñez A. Very brief training for laypeople in hands-only cardiopulmonary resuscitation. Effect of real-time feedback. The American Journal of Emergency Medicine. 2016;34:993–8.

## <18-year-old participants

1. Adelborg K, Thim T, Secher N, Grove EL, Løfgren B. Benefits and shortcomings of mandatory first aid and basic life support courses for learner drivers. Resuscitation. 2011;82:614–7.
2. Almousa O, Prates J, Yeslam N, Mac Gregor D, Zhang J, Phan V, et al. Virtual Reality Simulation Technology for Cardiopulmonary Resuscitation Training: An Innovative Hybrid System With Haptic Feedback. Simulation & Gaming. 2019;50:6–22.
3. Bakke HK, Steinvik T, Angell J, Wisborg T. A nationwide survey of first aid training and encounters in Norway. BMC Emerg Med. 2016;17:6.
4. Becker KJ, Fruin MS, Gooding TD, Tirschwell DL, Love PJ, Mankowski TM. Community-Based Education Improves Stroke Knowledge. Cerebrovasc Dis. 2001;11:34–43.
5. Cason CL, Stiller J. Performance outcomes of an online first aid and CPR course for laypersons. Health Education Journal. 2011;70:458–67.
6. Chang MP, Gent LM, Sweet M, Potts J, Ahtone J, Idris AH. A novel educational outreach approach to teach Hands-Only Cardiopulmonary Resuscitation to the public. Resuscitation. 2017;116:22–6.
7. Goolsby C, Branting A, Chen E, Mack E, Olsen C. Just-in-Time to Save Lives: A Pilot Study of Layperson Tourniquet Application. Acad Emerg Med. 2015;22:1113–7.
8. Harvey LA, Barr ML, Poulos RG, Finch CF, Sherker S, Harvey JG. A population‐based survey of knowledge of first aid for burns in New South Wales. Medical Journal of Australia. 2011;195:465–8.
9. Jelinek GA, Gennat H, Celenza T, O’Brien D, Jacobs I, Lynch D. Community attitudes towards performing cardiopulmonary resuscitation in Western Australia. Resuscitation. 2001;51:239–46.
10. Nielsen AM, Isbye DL, Lippert F, Rasmussen LS. Distributing personal resuscitation manikins in an untrained population: how well are basic life support skills acquired? Emerg Med J. 2012;29:587–91.
11. Nordberg M, Castrén M, Lindström V. Primary Trauma Triage Performed by Bystanders: An Observation Study. Prehosp Disaster med. 2016;31:353–7.
12. Pujalte-Jesús MJ, Leal-Costa C, Ruzafa-Martínez M, Ramos-Morcillo AJ, Díaz Agea JL. Relief Alternatives during Resuscitation: Instructions to Teach Bystanders. A Randomized Control Trial. IJERPH. 30. 2020;17:5495.
13. Marco CA, Larkin GL. Public education regarding resuscitation: Effects of a multimedia intervention. Annals of Emergency Medicine. 2003;42:256–60.
14. Regard S, Rosa D, Suppan M, Giangaspero C, Larribau R, Niquille M, et al. Evolution of Bystander Intention to Perform Resuscitation Since Last Training: Web-Based Survey. JMIR Form Res. 2020;4:e24798. doi:10.2196/24798.
15. Wallace HJ, O’Neill TB, Wood FM, Edgar DW, Rea SM. Determinants of burn first aid knowledge: Cross-sectional study. Burns. 2013;39:1162–9.
16. Wiese CH, Wilke H, Bahr J, Graf BM. Practical examination of bystanders performing Basic Life Support in Germany: a prospective manikin study. BMC Emerg Med. 2008;8:14.

## First aid of children

1. Savastano S, Vanni V. Cardiopulmonary resuscitation in real life: The most frequent fears of lay rescuers. Resuscitation. 2011;82:568–71.

## Elements of evaluation or study set up unclear

1. Birnbaum A, McBurnie MA, Powell J, Ottingham LV, Riegel B, Potts J, et al. Modeling instructor preferences for CPR and AED competence estimation. Resuscitation. 2005;64:333–9.
2. Guo N, Hampton MD. Improve Bystander Cardiopulmonary Resuscitation in the Chinese Community. Int Q Community Health Educ. 2021;41:309–14.
3. Kureckova V, Gabrhel V, Zamecnik P, Rezac P, Zaoral A, Hobl J. First aid as an important traffic safety factor – evaluation of the experience–based training. Eur Transp Res Rev. 2017;9:5.

## Specific elements of evaluation lacking

1. Barnhart JM. Awareness of Heart Attack Symptoms and Lifesaving Actions Among New York City Area Residents. Journal of Urban Health: Bulletin of the New York Academy of Medicine. 2005;82:207–15.
2. Chamberlain D, Smith A, Colquhoun M, Handley AJ, Kern KB, Woollard M. Randomised controlled trials of staged teaching for basic life support 2. Comparison of CPR performance and skill retention using either staged instruction or conventional training. Resuscitation. 2001;50: 27 –37.
3. Celik S. A media comparison study on first aid instruction. Health Education Journal. 2013;72:95–101.
4. Hawkes CA, Brown TP, Booth S, Fothergill RT, Siriwardena N, Zakaria S, et al. Attitudes to Cardiopulmonary Resuscitation and Defibrillator Use: A Survey of UK Adults in 2017. JAHA. 2019;8:e008267.
5. Ross EM, Redman TT, Mapp JG, Brown DJ, Tanaka K, Cooley CW, et al. Stop the Bleed: The Effect of Hemorrhage Control Education on Laypersons’ Willingness to Respond During a Traumatic Medical Emergency. Prehosp Disaster med. 2018;33:127–32.
6. Moon S, Ryoo HW, Ahn JY, Park JB, Lee DE, Kim JH, et al. A 5-year change of knowledge and willingness by sampled respondents to perform bystander cardiopulmonary resuscitation in a metropolitan city. PLoS ONE. 2019;14:e0211804.1.
7. Larsson EM, Mártensson NL, Alexanderson KAE. First-aid Training and Bystander Actions at Traffic Crashes — A Population Study. Prehosp Disaster med. 2002;17:134–41.
8. Lee MJ, Hwang SO, Cha KC, Cho GC, Yang HJ, Rho TH. Influence of nationwide policy on citizens’ awareness and willingness to perform bystander cardiopulmonary resuscitation. Resuscitation. 2013;84:889–94.
9. Richman PB, Bobrow BJ, Clark L, Noelck N, Sanders AB. Ability of Citizens in a Senior Living Community to Perform Lifesaving Cardiac Skills and Appropriately Utilize AEDs. The Journal of Emergency Medicine. 2007;33:395–9.
10. Ross EM, Redman TT, Mapp JG, Brown DJ, Tanaka K, Cooley CW, et al. Stop the Bleed: The Effect of Hemorrhage Control Education on Laypersons’ Willingness to Respond During a Traumatic Medical Emergency. Prehosp Disaster med. 2018;33:127–32.
11. Son JW, Ryoo HW, Moon S, Kim J, Ahn JY, Park JB, et al. Association between public cardiopulmonary resuscitation education and the willingness to perform bystander cardiopulmonary resuscitation: a metropolitan citywide survey. Clin Exp Emerg Med. 2017;4:80–7.
12. Van de Velde S, Roex A, Vangronsveld K, Niezink L, Van Praet K, Heselmans A, ym. Can training improve laypersons helping behaviour in first aid? A randomised controlled deception trial. Emerg Med J. 2013;30:292–7.
13. Xu Y, Li J, Wu Y, Yue P, Wu F, Xu Y. An audio-visual review model enhanced one-year retention of cardiopulmonary resuscitation skills and knowledge: A randomized controlled trial. International Journal of Nursing Studies. helmikuuta 2020;102:103451.

## Qualitative study

1. Arbon P, Hayes J, Woodman R. First Aid and Harm Minimization for Victims of Road Trauma: A Population Study. Prehosp Disaster med. 2011;26:276–82.
2. Dobbie F, Uny I, Eadie D, Duncan E, Stead M, Bauld L, et al. Barriers to bystander CPR in deprived communities: Findings from a qualitative study. Lazzeri C, toimittaja. PLoS ONE. 10. 2020;15:e0233675. doi: 10.1371/journal.pone.0233675.
3. Kamışlı H, Özonur M. The Effects of Training – Based on Knowles’ Adult Education Principles – On Participants. EURASIA J Mathematics, Science and Technology Education. 2017;13:8405–8414.
4. Kulnik ST, Halter M, Hilton A, Baron A, Garner S, Jarman H, ym. Confidence and willingness among laypersons in the UK to act in a head injury situation: a qualitative focus group study. BMJ Open. marraskuuta 2019;9:e033531. doi:10.1136/ bmjopen-2019-033531.
5. Riegel B, Mosesso VN, Birnbaum A, Bosken L, Evans LM, Feeny D, et al. Stress reactions and perceived difficulties of lay responders to a medical emergency. Resuscitation. 2006;70:98–106.
6. Sasson C, Haukoos JS, Bond C, Rabe M, Colbert SH, King R, et al. Barriers and Facilitators to Learning and Performing Cardiopulmonary Resuscitation in Neighborhoods With Low Bystander Cardiopulmonary Resuscitation Prevalence and High Rates of Cardiac Arrest in Columbus, OH. Circ: Cardiovascular Quality and Outcomes. 2013;6:550–8.
7. Yeung J, Meeks R, Edelson D, Gao F, Soar J, Perkins GD. The use of CPR feedback/prompt devices during training and CPR performance: A systematic review. Resuscitation. 2009;80:743–51.

## No follow guidelines (ERC)

1. Anderson GS, Gaetz M, Statz C. CPR Skill Retention of First Aid Attendants within the Workplace. Prehosp Disaster med. 2012;27:312–8.
2. Chung C, Siu AY, Po LL, Lam C, Wong PC. Comparing the effectiveness of video self-instruction versus traditional classroom instruction targeted at cardiopulmonary resuscitation skills for laypersons: a prospective randomised controlled trial. Hong Kong Med J 2010;16:165-70.
3. Ireland CJ, Zeitz KM, Bridgewater FHG. Acquiring and Maintaining Competence in the Application of Extrication Cervical Collars by a Group of First Responders. Prehosp Disaster med. 2008;23:530–6.

## Medical setup

1. Neset A, Birkenes TS, Furunes T, Myklebust He, Mykletun RJ, Odegaard S, et al. A randomized trial on elderly laypersons’ CPR performance in a realistic cardiac arrest simulation: Elderly laypersons’ CPR performance. Acta Anaesthesiol Scand. 2012;56:124–31.
2. O’Callaghan G, Murphy S, Loane D, Farrelly E, Horgan F. Stroke Knowledge in an Irish Semi-Rural Community-Dwelling Cohort and Impact of a Brief Education Session. Journal of Stroke and Cerebrovascular Diseases. 2012;21:629–38.

## Evaluation method did no assess first aid skills after training

1. Bray JE, Smith K, Case R, Cartledge S, Straney L, Finn J. Public cardiopulmonary resuscitation training rates and awareness of hands-only cardiopulmonary resuscitation: a cross-sectional survey of Victorians. Emergency Medicine Australasia. 2017;29:158–64.
2. Fogle CC, Oser CS, Troutman TP, McNamara M, Williamson AP, Keller M, et al. Public Education Strategies to Increase Awareness of Stroke Warning Signs and the Need to Call 911. Journal of Public Health Management and Practice. 2008;14:e17–22.
3. Lippmann J, Livingston P, Craike MJ. Comparison of two modes of delivery of first aid training including basic life support. Health Education Journal. 2011;70:131–40.
4. Neset A, Birkenes TS, Furunes T, Myklebust He, Mykletun RJ, Odegaard S, et al. A randomized trial on elderly laypersons’ CPR performance in a realistic cardiac arrest simulation: Elderly laypersons’ CPR performance. Acta Anaesthesiol Scand. 2012;56:124–31.
5. O’Callaghan G, Murphy S, Loane D, Farrelly E, Horgan F. Stroke Knowledge in an Irish Semi-Rural Community-Dwelling Cohort and Impact of a Brief Education Session. Journal of Stroke and Cerebrovascular Diseases. 2012;21:629–38.
6. Silver FL, Rubini F, Black D, Hodgson CS. Advertising Strategies to Increase Public Knowledge of the Warning Signs of Stroke. Stroke. 2003;34:1965–8. doi: 10.1161/01.STR.0000083175.01126.62.
7. Sipsma K, Stubbs BA, Plorde M. Training rates and willingness to perform CPR in King County, Washington: A community survey. Resuscitation. 2011;82:564–7.
8. Thierbach AR, Pelinka LE, Reuter S, Mauritz W. Comparison of bystander trauma care for moderate versus severe injury. Resuscitation. 2004; 60: 271-277.
