# Supplementary material for: How to evaluate first aid skills after training: a systematic review
Source: Scand J Trauma Resusc Emerg Med. 2022 Nov 8;30:56. doi: 10.1186/s13049-022-01043-z (PMC9641962; doi:10.1186/s13049-022-01043-z)
Supplement: Supplementary file 3 — Supplementary Material 3 [file 13049_2022_1043_MOESM3_ESM.docx]

**Additional file 3. Integrated CPR studies**

1. Ahn JY, Cho GC, Shon YD, Park SM, Kang KH. Effect of a reminder video using a mobile phone on the retention of CPR and AED skills in lay responders. Resuscitation. 2011;82:1543-1547.
2. Baldi E, Cornara S, Contri E, Epis F, Fina D, Zelaschi B. et al. Real-time visual feedback during training improves laypersons’ CPR quality: A randomized controlled manikin study. Canadian journal of emergency medicine. 2017;19:480-487.
3. Benoit JL, Vogele J, Hart KW, Lindsell CJ, McMullan JT. Passive ultra-brief video training improves performance of compression-only cardiopulmonary resuscitation. Resuscitation 2017;115:116-119.
4. Beskind DL, Stolz U, Thiede R, Hoyer R, Robertson W, Brown J. et al. Viewing an ultra-brief chest compression only video improves some measures of bystander CPR performance and responsiveness at a mass gathering event. Resuscitation 2017;118:96-100.
5. Birkenes TS, Myklebust H, Neset A, Kramer-Johansen J. Quality of CPR performed by trained bystanders with optimized pre-arrival instructions. Resuscitation 2013;85:124-130.
6. Birkenes TS, Myklebust H, Neset A, Olasveengen TM, Kramer-Johansen J. Video analysis of dispatcher–rescuer teamwork–Effects on CPR technique and performance. Resuscitation 2011;83:494-499.
7. Bobrow BJ, Vadeboncoeur TF, Spaite DW, Potts J, Denninghoff K, Chikani V. et al. The effectiveness of ultrabrief and brief educational videos for training lay responders in hands-only cardiopulmonary resuscitation: Implications for the future of citizen cardiopulmonary resuscitation training. Circulation Cardiovascular quality and outcomes 2011;4:220-226.
8. Boet S, Bould MD, Pigford A-A, Rössler B, Nambyiah P. Retention of basic life support in laypeople: Mastery learning vs. time-based education. Prehospital emergency care 2017;21:362-377.
9. Brinkrolf P, Bohn A, Lukas R-P, Heyse M, Dierschke T, Van Aken HK. et al. Senior citizens as rescuers: Is reduced knowledge the reason for omitted lay-resuscitation-attempts? Results from a representative survey with 2004 interviews. PloS ONE. 2017;12:e0178938. doi: [10.1371/journal.pone.0178938](https://doi.org/10.1371/journal.pone.0178938).
10. Celenza T, Gennat HC, O`Brien, Jacobs IG, Lynch DM, Jelinek GA. Community competence in cardiopulmonary resuscitation. Resuscitation. 2002;55:157.
11. Chamberlain D, Smith A, Woollard M, Colquhoun M, Handley AJ, Leaves S. et al. Trials of teaching methods in basic life support (3):: Comparison of simulated CPR performance after first training and at 6 months, with a note on the value of re-training. Resuscitation 2002;53:179-187.
12. Choa M, Cho J, Choi YH, Kim S, Sung JM, Chung HS. Animation-assisted CPRII program as a reminder tool in achieving effective one-person-CPR performance. Resuscitation. 2009;80:680-684.
13. Choa M, Park I, Chung HS, Yoo SK, Shim H, Kim S. The effectiveness of cardiopulmonary resuscitation instruction: Animation versus dispatcher through a cellular phone. Resuscitation 2007;77:87-94.
14. Choi HJ, Lee CC, Lim TH, Kang BS, Singer AJ, Henry MC. Effectiveness of mouth-to-mouth ventilation after video self-instruction training in laypersons. The American journal of emergency medicine. 2010;28:654-657.
15. Christenson J, Nafziger S, Compton S, Vijayaraghavan K, Slater B, Ledingham B, et al. The effect of time on CPR and automated external defibrillator skills in the public access defibrillation trial. Resuscitation. 2006;74:52-62.
16. Chung CH, Siu AYC, Po LLK, Lam CY & Wong PCY. Comparing the effectiveness of video self-instruction versus traditional classroom instruction targeted at cardiopulmonary resuscitation skills for laypersons: A prospective randomised controlled trial. Hong Kong Med J. 2010;16:165-70.
17. de Vries W. & Handley AJ. A web-based micro-simulation program for self-learning BLS skills and the use of an AED. Resuscitation. 2007;75:491-498.
18. de Vries W, Turner NM, Monsieurs KG, Bierens, Joost J. L. M, Koster RW. Comparison of instructor-led automated external defibrillation training and three alternative DVD-based training methods. Resuscitation. 2010;81:1004-1009.
19. Eaton G, Renshaw J, Gregory P, Kilner T. Can the british heart foundation PocketCPR application improve the performance of chest compressions during bystander resuscitation: A randomised crossover manikin study. Health informatics journal. 2018;24:14-23.
20. Einspruch EL, Lynch B, Aufderheide TP, Nichol G, Becker L. Retention of CPR skills learned in a traditional AHA heartsaver course versus 30-min video self-training: A controlled randomized study. Resuscitation. 2007;74:476-486.
21. Ettl F, Testori C, Weiser C, Fleischhackl S, Mayer-Stickler, Herkner H, et al. Updated teaching techniques improve CPR performance measures: A cluster randomized, controlled trial. Resuscitation. 2011;82:730-735.
22. Gruenerbl A, Pirkl G, Monger E, Gobbi M, Lukowicz P. Smart-watch life saver. Proceedings of the 2015 ACM International Symposium on wearable computers. ISWC. 2015:19-26.
23. Hafner JF, Andrew C. Huaping Wang J, Bleess BB, Tham SK. Death Before Disco: The Effectiveness of a Musical Metronome in Layperson Cardiopulmonary Resuscitation Training. The Journal of Emergency Medicinh. 2015; 48:43-52.
24. Harve H, Jokela J, Tissari A, Saukko A, Okkolin T, Pettilä V, et al. Defibrillation and the quality of layperson cardiopulmonary resuscitation—Dispatcher assistance or training? Resuscitation. 2008;80:275-277.
25. Hsu S, Kuo C, Weng Y, Lin C, Chen J. The effectiveness of teaching chest compression first in a standardized public cardiopulmonary resuscitation training program. Medicine. 2019;98.pe14418.
26. Isbye DL, Rasmussen LS, Lippert FK, Rudolph SF, Ringsted CV. Laypersons may learn basic life support in 24min using a personal resuscitation manikin. Resuscitation. 2006;69:435-442.
27. Jarrah S, Judeh M, Aburuz ME. Evaluation of public awareness, knowledge and attitudes towards basic life support: A cross-sectional study. BMC Emerg Med. 2018;18:37.
28. Jensen TW, Møller TP, Viereck S, Hansen JR, Pedersen TE, Ersbøll AK, al. A nationwide investigation of CPR courses, books, and skill retention. Resuscitation. 2019;134:110-121.
29. Jiang Y, Wu B, Long L, Li J, Jin X. Attitudes and willingness toward out-of-hospital cardiopulmonary resuscitation: A questionnaire study among the public trained online in china. BMJ Open. 2020;10:e038712.
30. Jones I, Handley AJ, Whitfield R, Newcombe R, Chamberlain D. A preliminary feasibility study of a short DVD-based distance-learning package for basic life support. Resuscitation. 2007;75:350-356.
31. Ko RJM, Lim SH, Wu VX, Leong TY, Liaw SY. Easy-to-learn cardiopulmonary resuscitation training programme: A randomised controlled trial on laypeople's resuscitation performance. Singapore medical journal. 2017;59:217-223.
32. Konstandinos HD, Evangelos KI, Stamatia K, Thyresia S, Zacharenia AD. Community cardiopulmonary resuscitation training in greece. Research in nursing &amp; health. 2008;3:165-171.
33. Leary M, Almodovar A, Buckler D, Bhardwaj A, Blewer A, Abella B. Using an immersive virtual reality system to assess lay provider response to an unannounced simulated sudden cardiac arrest in the out-of-hospital setting. Simulation in healthcare : journal of the Society for Medical Simulation. 2019;14:82-89.
34. Lund-Kordahl I, Mathiassen M, Melau J, Olasveengen TM, Sunde K, Fredriksen K. Relationship between level of CPR training, self-reported skills, and actual manikin test performance—an observational study. Int J Emerg Med. 2019;12:1-8.
35. Lynch B, Einspruch EL. With or without an instructor, brief exposure to CPR training produces significant attitude change. Resuscitation. 2010;81:568-575.
36. Lynch B, Einspruch EL, Nichol G, Aufderheide TP. Assessment of BLS skills: Optimizing use of instructor and manikin measures. Resuscitation. 2007;76:233-243.
37. Lynch B, Einspruch EL, Nichol G, Becker LB, Aufderheide TP, Idris A. Effectiveness of a 30-min CPR self-instruction program for lay responders: A controlled randomized study. Resuscitation. 2005;67:31-43.
38. Maaß S, Sense F, Gluck K, van Rijn H. Keeping bystanders active: Resuscitating resuscitation skills. Frontiers in public health. 2019;7:177.
39. Mahony PH, Griffiths RF, Larsen P, Powell D. Retention of knowledge and skills in first aid and resuscitation by airline cabin crew. Resuscitation. 2007;76:413-418.
40. Mancini ME, Ne-Bc M, Cazzell S, Kardong-Edgren, Cason CL. Improving workplace safety training using a self-directed CPR-AED learning program. AAOHN J. 2009;57:159-67;168-9.
41. Meischke HW, Rea T, Eisenberg MS, Schaeffer SM, Kudenchuk P. Training seniors in the operation of an automated external defibrillator: A randomized trial comparing two training methods. Annals of emergency medicine. 2001;38:216-222.
42. Merchant RM, Abella BS, MPhil, Abotsi EJ, Smith TM, Long JA, et al. Cell phone cardiopulmonary resuscitation: Audio instructions when needed by lay rescuers: A randomized, controlled trial. Annals of emergency medicine. 2009;55:538-543.
43. Miller B & Pellegrino JL. Measuring Intent to Aid of Lay Responders: Survey Development and Validation. Health Educ Behav. 2018;45:730–40.
44. Nebsbjerg MA, Rasmussen SE, Bomholt KB, Krogh LQ, Krogh K, Povlsen JA, et al. Skills among young and elderly laypersons during simulated dispatcher assisted CPR and after CPR training. Acta Anaesthesiol Scand. 2017;62:125.
45. Nishiyama C, Iwami T, Kawamura T, Ando M, Kajino K, Yonemoto N, et al. Effectiveness of simplified chest compression-only CPR training program with or without preparatory self-learning video: A randomized controlled trial. Resuscitation. 2009;80:1164-1168. doi: 10.1016/j.resuscitation.2009.06.019.
46. Paal P, Pircher I, Baur T, Gruber E, Strasak AM, Herff H, et al. Mobile phone-assisted basic life support augmented with a Metronome. The Journal of emergency medicine. 2012;43:472-477.
47. Papalexopoulou K, Chalkias A, Dontas J, Pliatsika P, Giannakakos C, Papapanagiotou P, et al. Education and age affect skill acquisition and retention in lay rescuers after a european resuscitation council CPR/AED course. Heart&Lung. 2014;43:66-71.
48. Plata C, Stolz M, Warnecke T, Steinhauser S, Hinkelbein J, Wetsch WA, et al. Using a smartphone application (PocketCPR) to determine CPR quality in a bystander CPR scenario — A manikin trial. Resuscitation. 2019;137:87-93.
49. Roppolo LP, Pepe PE, Campbell L, Ohman K, Kulkarni H, Miller R, et al. Prospective, randomized trial of the effectiveness and retention of 30-min layperson training for cardiopulmonary resuscitation and automated external defibrillators: The american airlines study. Resuscitation. 2007;74:276-285.
50. Schiefer JL, Schuller H, Fuchs PC, Bagheri M, Grigutsch D, Klein M, et al. Basic life support knowledge in Germany and the influences of demographic factors. PloS ONE. 2020;15:e0237751. doi: 10.1371/journal.pone.0237751.
51. Sopka S, Biermann H, Rossaint R, Rex S, Jäger M, Skorning M, et al. Resuscitation training in small-group setting--gender matters. Scandinavian journal of trauma, resuscitation and emergency medicine. 2013;21:30.
52. Wik L, Myklebust H, Auestad BH, Steen PA. Retention of basic life support skills 6 months after training with an automated voice advisory manikin system without instructor involvement. Resuscitation. 2002;52:273.
53. Wik L, Myklebust H, Auestad BH, Steen PA. Twelve-month retention of CPR skills with automatic correcting verbal feedback. Resuscitation. 2005;66:27-30.
54. Woollard M, Smith A, Whitfield R, Chamberlain D, West R, Newcombe R, et al. To blow or not to blow: A randomised controlled trial of compression-only and standard telephone CPR instructions in simulated cardiac arrest. Resuscitation. 2003;59:123.
55. Woollard M, Whitfield R, Smith A, Colquhoun M, Newcombe RG, Vetter N, et al. Skill acquisition and retention in automated external defibrillator (AED) use and CPR by lay responders: A prospective study. Resuscitation. 2004;60:17-28.
